# B2 disorder-driven magnetic properties of hydrothermally synthesized Fe–Co–Al full Heusler alloys: a combined theoretical and experimental study on bulk and disordered nanoparticles

**DOI:** 10.1039/d6na00296j

**Published:** 2026-06-02

**Authors:** Abegail, Riyajul Islam, C. Borgohain, J. P. Borah

**Affiliations:** a Department of Physics, National Institute of Technology Nagaland 797103 India jpborah@rediffmail.com; b Central Instrument Facility (CIF), Indian Institute of Technology Guwahati 784029 India

## Abstract

A series of Fe_2−*x*_Co_1+*x*_Al full Heusler alloy nanoparticles were successfully prepared using a controlled hydrothermal technique with particle sizes ranging between 16 and 35 nm. First-principles DFT calculations were also performed to predict the electronic structures, magnetic moments, and phase stability across various compositions. The structure and morphology of the nanoparticles were studied using X-rays and the electron diffraction technique, which validated the presence of chemical disorder of B2 type in the prepared alloys. The structural and magnetic properties of the alloys with varied Fe/Co ratios were studied with optimized pH and a reducing agent. The experimentally obtained moments are found to be larger than the predicted L2_1_-based Slater–Pauling values of 4.0*µ*_B_ f.u.^−1^ and 5.00*µ*_B_ f.u.^−1^ for Fe_2_CoAl and Co_2_FeAl respectively. The primary factor influencing the magnetic behavior of the alloy system has been attributed to the presence of secondary phases and the chemical disorder. This work provides a simple hydrothermal synthesis method to prepare B2 disordered full Heusler alloy nanoparticles and shows that compositional control is a viable approach towards improving saturation magnetization and other magnetic parameters in these promising materials for spintronic applications.

## Abstract 2

A series of Fe_2−*x*_Co_1+*x*_Al full Heusler alloy nanoparticles were successfully prepared using a controlled hydrothermal technique with particle sizes ranging between 16 and 35 nm.

## Graphical abstract

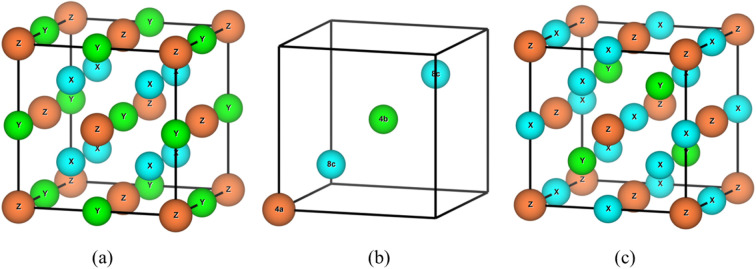

## 1 Introduction

Heusler alloys (HAs), a class of intermetallic alloys, have gained immense attention in the last few decades due to their applicability in a wide range of fields that include spintronic devices,^1–3^ thermoelectric materials,^4,5^ magnetocaloric refrigeration,^6^ magnetic shape memory effects,^7,8^ and topological insulators.^9,10^ They have a remarkable history; discovered in 1903, Friedrich Heusler successfully synthesized a ferromagnetic alloy from two parts of copper and one part each of manganese and aluminum (Cu_2_MnAl), where none of its constituents were ferromagnetic themselves. Today, the Heusler family extends to more than 1500 compounds, studied for a broad range of multi-functionalities.^11^ High spin polarization in magnetic materials is the cornerstone of the performance of spintronic devices. Most Full Heusler Alloys (FHAs) exhibit 100% spin polarization at the Fermi level (*E*_F_), *i.e.*, one of the spin channels is conducting while the other is semi-conducting with a band gap. This unavailability of electronic states at *E*_F_ minimizes the energy loss by spin-flip scattering, resulting in a lower Gilbert damping constant (*α*). However, there are reports of reduced spin polarization in some HAs, mainly attributed to their structural disorder,^12–14^ strain,^13^ and also the surface effect.^15^ Heusler alloys are also extensively studied because of their high magnetic moment and exceptionally high Curie temperature (*T*_c_). Literature reports a high saturation magnetization (*M*_s_) of 6.5*µ*_B_ f.u.^−1^ and *T*_c_ ∼ 1262 K for disordered Co_2_FeAl,^16^*M*_s_ ∼ 6.5*µ*_B_ f.u.^−1^ and *T*_c_ ∼ 1261 K for Co_2_FeAl nanoparticles having a mean size of 16 ± 10 nm,^17^ and *M*_s_ ∼ 6.7 ± 0.1*µ*_B_ f.u.^−1^ with *T*_c_ ∼ 1135 ± 5 K for single phase Co_2_Fe_1.25_Ge_0.75_.^18^ Another characteristic feature of HAs is the linear dependence of the magnetic moment on the number of valence electrons.^19,20^ Above all, the easily tunable nature of these materials by adapting different synthesis conditions,^21–23^ doping/substitution,^24,25^ inducing site/anti-site disorder,^26–28^ defects,^29,30^ and strains,^31,32^ is one of the main reasons why they have gained much attention in the field of research. Though these alloys are widely studied, preparing magnetic nanoparticles remains a challenge owing to their vulnerability to oxidation and positional disorder of the constituent elements.^33^

Although substantial progress has been made, there are still serious obstacles that need to be overcome before Heusler alloy nanoparticles can be used in practical applications. First, traditional synthesis approaches like arc melting, mechanical alloying, and electrospinning generally demand high temperatures (>800 °C), which usually cause uncontrolled grain growth, inconsistent compositional homogeneity, and wide particle size distributions.^34^ The oxygen affinity of constituent transition elements poses significant problems at the interfaces and degrades the intrinsic magnetic properties.^35^ Secondly, experimental studies aimed at systematically correlating off-stoichiometric Fe/Co ratios with structural ordering (L2_1_*vs.* B2 *vs.* A2 phases), morphology, and magnetic properties at the nanoscale have rarely been explored. Most studies focus mainly on Co-rich HAs;^36–39^ the effects of systematic compositional variations across the Fe_2−*x*_Co_1+*x*_Al series are relatively unexplored at the nanoparticle level. Studies on low-temperature wet-chemical synthesis methods show potential to address these issues, but are mostly unexplored for Fe–Co–Al Heusler systems. Among these methods, hydrothermal synthesis can especially offer controlled morphology, lower oxidation due to reduced processing temperatures, and better stoichiometry control.^40^ However, obtaining pure phase and ordered Heusler structures *via* hydrothermal methods is relatively difficult, especially for ternary systems, as all the synthesis parameters need to be fully optimized. The very fundamental question is whether it will be possible to achieve ordered Heusler nanoparticles by using low-temperature synthesis approaches with magnetic properties comparable to conventionally prepared materials while overcoming the oxidation challenges. To fill these gaps, there is a need for a combined theoretical–experimental approach that can study the composition effect and, simultaneously, validate structure–property relationships in the compounds. Indeed, it is this kind of integrated investigation that represents the pathway toward understanding correlations among composition, structure, and properties and would allow the rational design of Heusler nanoparticles for practical applications.

In this work, composition-dependent electronic, structural, and magnetic properties of Fe_2−*x*_Co_1+*x*_Al alloys have been studied both theoretically and experimentally. First-principles DFT calculations were conducted to predict electronic structures, magnetic moments, and phase stability across various compositions. The Fe_2−*x*_Co_1+*x*_Al alloys were then prepared by a facile one-step hydrothermal method in a Teflon-lined autoclave at a relatively low temperature (160 °C). The morphology and microstructure, phase purity, atomic ordering, and magnetic properties of the alloys with variable Fe/Co ratios and their relationship have been systematically investigated. This comprehensive study provides fundamental insights into how compositional tuning influences structural ordering and magnetic behavior in hydrothermally synthesized Heusler nanoparticles while demonstrating an energy-efficient synthesis route that could enable practical applications in spintronics, magnetic data storage, and other technologies where conventional high-temperature processing is impractical.

## 2 Materials and methods

### 2.1 Computational details and crystal structures

**Fig. 1:**
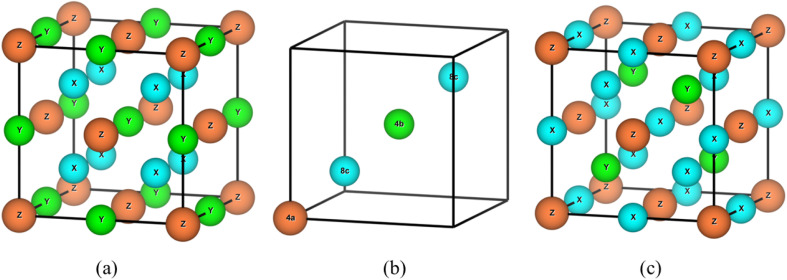
(a) L2_1_ crystal structure, (b) Wyckoff positions for the L2_1_ structure, and (c) XA inverse crystal structure of full Heusler alloys.

The X_2_YZ-type Full Heusler Alloy (FHA) crystallizes in either the ordered L2_1_ or XA structure, where X and Y are transition metals and Z is a p-block element. Both the L2_1_ and XA structures consist of four interpenetrating face-centered cubic sublattices (Fig. 1), with the X elements occupying two of these sublattices. The L2_1_ X_2_YZ full Heusler alloy has a cubic structure with the space group *Fm*3̄*m* (space group no. 225), where X atoms occupy the Wyckoff positions 8c (1/2, 1/2, 1/2) & (3/4, 3/4, 3/4), while Y atoms occupy 4b (1/2, 1/2, 1/2) and Z atoms at 4a (0, 0, 0). The inverse XA also exhibits a cubic structure with the space group *F*4̄3*m* (space group no. 216). This structure is typically favored when the Y element has a higher atomic number than the X element, belonging to the same period. In the XA structure, the X atoms occupy 4b (1/2, 1/2, 1/2) and one of the 8c positions, *i.e.*, (3/4, 3/4, 3/4), while the Y and Z atoms occupy the other 8c (1/4, 1/4, 1/4) & 4a (0, 0, 0) positions. This classification is based on the electronegativity of the elements,^41^ where the XA inverse structure is favored for compounds in which the Y element is more electronegative than the X element.

The ground state calculations to investigate the structural and magnetic properties of Fe_2−*x*_Co_1+*x*_Al alloys were performed using the Density Functional Theory (DFT) using the Vienna *Ab initio* Simulation Package (VASP),^42^ which is based on the Projector Augmented Wave (PAW) method.^43^ The Perdew–Burke–Ernzerhof (PBE) exchange–correlation functional was utilized within the purview of the Generalized Gradient Approximation (GGA)^44^ for all the calculations, since Fe–Co-based Heusler systems are moderately correlated, and previous literature^17^ reports consistent results using the GGA functional. A plane-wave basis set with a cut-off energy of 600 eV was set with a force convergence limit below 0.01 eV Å^−1^ and a convergence threshold of 10^−6^ eV. In a single unit cell, there are a total of 16 atoms, *i.e.*, for Fe_2_CoAl (8Fe atoms, 4Co atoms, and 4Al atoms). Consequently, the subsequent off-stoichiometric composition in the Fe_2−*x*_Co_1+*x*_Al series was modeled systematically by substituting Fe atoms with Co atoms at the crystallographically equivalent Fe sites while maintaining the Al sublattice unchanged. To confirm the ferromagnetic ground state, we considered both the antiferromagnetic (AFM) and ferromagnetic (FM) spin configurations, and the FM state yielded a lower formation energy than the AFM state for all the compositions. To relax the crystal structures and obtain electronic structure calculations, a *k*-point mesh of 10 × 10 × 10 Monkhorst–Pack was considered,^45^ and optimization was performed using energy minimization.

### 2.2 Experimental details

**Fig. 2:**
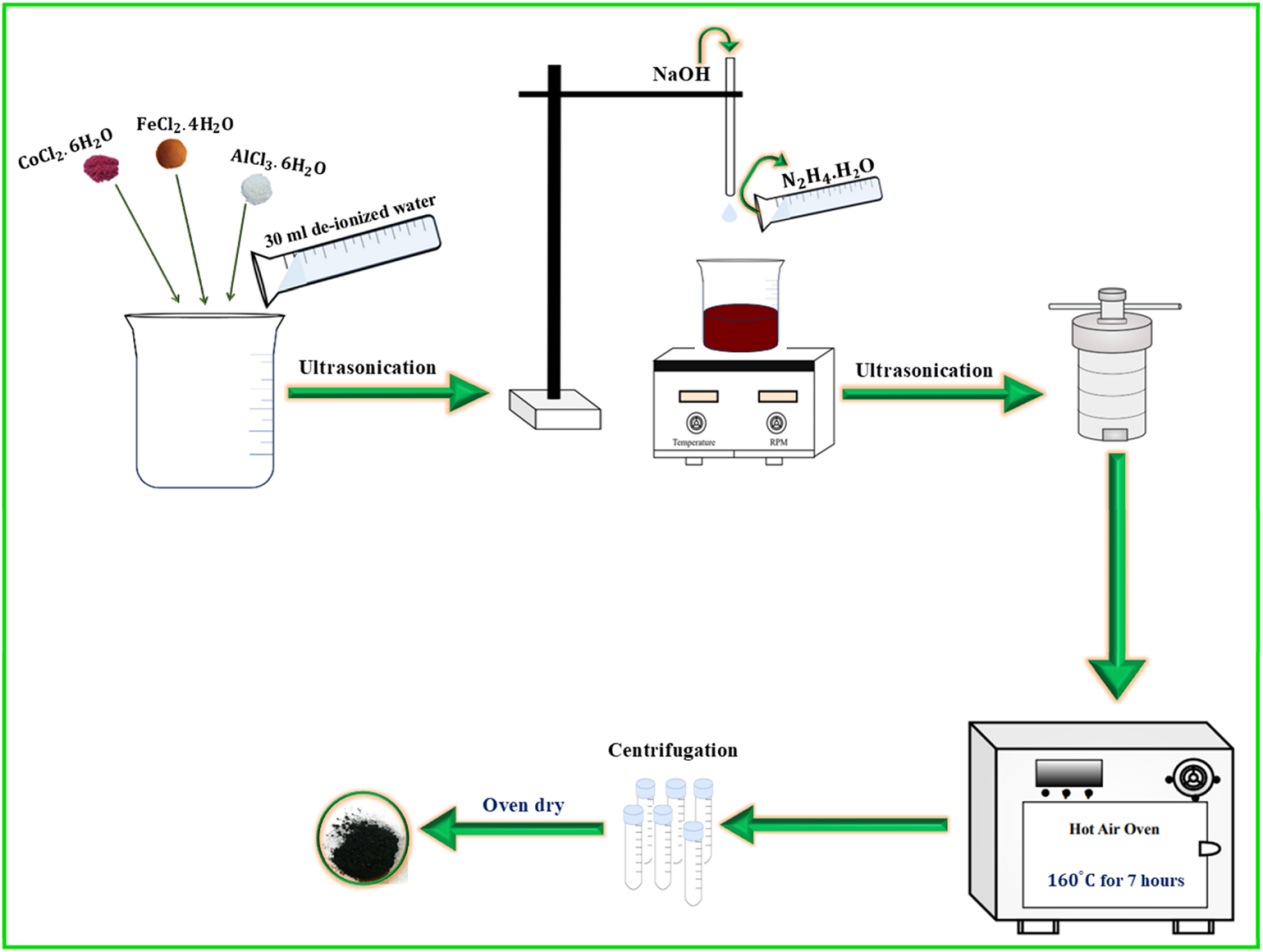
Schematic diagram of the synthesis process for the preparation of FCA–CFA alloys.

A series of Fe_2−*x*_Co_1+*x*_Al (FCA–CFA) nanoparticles were prepared using the hydrothermal method. The composition included Fe_2_CoAl (FCA), Co_1.2_Fe_1.8_Al, Co_1.4_Fe_1.6_Al, Co_1.6_Fe_1.4_Al, Co_1.8_Fe_1.2_Al, and Co_2_FeAl (CFA). The precursors used were cobalt chloride hexahydrate (CoCl_2_·6H_2_O), iron chloride tetrahydrate (FeCl_2_·4H_2_O), and aluminum chloride hexahydrate (AlCl_3_·6H_2_O). For synthesizing Fe_2_CoAl (FCA), 10 mmol FeCl_2_·4H_2_O, 5 mmol CoCl_2_·6H_2_O, and 5 mmol AlCl_3_·6H_2_O were dissolved in 30 mL deionized water and ultrasonicated for 10 minutes. With the precursor solution under magnetic stirring, a NaOH solution was added dropwise to maintain alkaline conditions. The mixture was then ultrasonically treated for 10 minutes. Next, 10 mL of hydrazine hydrate (N_2_H_4_·H_2_O) was added as a reducing agent, followed by another round of ultrasonication. The mixed solution was treated at 160 °C for 7 h, after which it was washed multiple times with ethanol and deionized water to remove soluble impurities and by-products. Finally, the sample was air-dried at 50 °C for 8 h. Similarly, by varying the Fe/Co molar ratios, Co_1.2_Fe_1.8_Al, Co_1.4_Fe_1.6_Al, Co_1.6_Fe_1.4_Al, Co_1.8_Fe_1.2_Al, and Co_2_FeAl (CFA) were prepared. Fig. 2 presents the described synthesis process.

The formation of the CFA/FCA alloy is dependent on a strong alkaline environment, which greatly enhances the reducing ability of the hydrazine hydrate. The excess OH^−^ significantly influences the reduction potentials of the Fe^2+^/Fe, Co^2+^/Co, and Al^3+^/Al redox pairs. This shifts the potentials closer together, enabling the ions to be easily co-reduced into their respective metallic atoms and initiating the generation of the alloy nuclei. The chemical reaction for CFA can be described as follows:2Co^2+^ + Fe^2+^ + Al^3+^ + 9OH^−^ = Co(OH)_2_↓ + Fe(OH)_2_↓ + Al(OH)_3_↓8Co(OH)_2_↓ + 4Fe(OH)_2_↓ + 4Al(OH)_3_↓ + 9N_2_H_4_·H_2_O = 4Co_2_FeAl + 9N_2_↑ + 45H_2_O

### 2.3 Characterization

The crystallographic phase of the samples was characterized by X-ray diffraction (Rigaku, Ultima IV) with CuKα radiation (*λ* = 1.5406 Å). A high-resolution transmission electron microscope (HRTEM: JEOL, model: JEM 2100) was utilized to observe the morphology and to estimate the average particle size of the samples. The magnetic properties of the synthesized alloys were characterized using a Vibrating Sample Magnetometer (VSM: Lake Shore, model: 7410 series).

## 3 Results and discussion

### 3.1 DFT analysis

The structural stability of the compound is validated using its formation energy, which represents the excess energy of the compound relative to its constituent elements. The enthalpy of formation energy (*H*_f_) for the compounds was calculated using the following relation (below) and is tabulated in Table 1.

1 *H*_f_(X_2_YZ) = *E*(X_2_YZ) − [2*E*(X) + *E*(Y) + *E*(Z)]

where *E*(X_2_YZ) is the total energy per formula unit and *E*(X), *E*(Y), and *E*(Z) denote the ground-state total energy per atom in their stable bulk phases of the X, Y, and Z atoms, respectively. All compounds under investigation exhibit a negative *H*_f_, confirming their structural stability. This stability is most pronounced, *i.e.*, most negative for Co_2_FeAl (*H*_f_ = −5.64 eV per atom), and the value systematically increases (becomes less negative) with increasing Fe content, indicating a slight reduction of stability.

**Table 1:** Calculated optimized lattice constants, volume, symmetry, formation energies, and average magnetic moments of atoms at individual sites, along with the total magnetic moment *M*_total_ per formula unit of the compounds

Compound	Optimized lattice constants (Å)			Symmetry (space group)	Volume (Å^3^)	Formation energy, *H*_f_ (eV per atom)	*m* ^Co^_s_ (*µ*_B_ f.u.^−1^)	*m* ^Fe^_s_ (*µ*_B_ f.u.^−1^)	*m* ^Al^_s_ (*µ*_B_ f.u.^−1^)	*M*_total_ (*µ*_B_ f.u.^−1^)
	*a*	*b*	*c*							
Fe_2_CoAl	5.699	5.699	5.699	216 (*F*4̄3*m*)	185.09	−4.27	1.04	2.07	−0.048	5.13
Co_1.25_Fe_1.75_Al	5.717	5.717	5.656	111 (*P*4̄2*m*)	184.86	−4.64	1.02	2.17	−0.045	5.04
Co_1.5_Fe_1.5_Al	5.688	5.688	5.688	224 (*Pn*3̄*m*)	184.02	−4.95	1.02	2.22	−0.040	4.84
Co_1.75_Fe_1.25_Al	5.690	5.690	5.690	215 (*P*4̄3*m*)	184.22	−5.26	1.07	2.45	−0.037	4.91
Co_2_FeAl	5.697	5.697	5.697	225 (*Fm*3̄*m*)	184.90	−5.64	1.18	2.76	−0.033	5.09

**Fig. 3:**
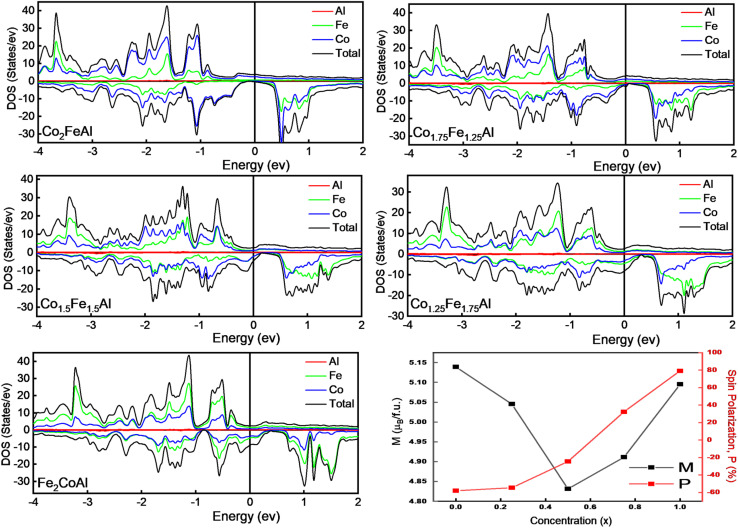
Spin-polarized total and partial DOS of each element of Fe_2−*x*_Co_1+*x*_Al compounds near *E*_F_, and variation of spin polarization and spin magnetic moment with increasing *x*.

We next investigate how the different compositions influence the density of states (DOS), focusing specifically on the vicinity of the Fermi level (*E*_F_). Fig. 3 shows the spin-polarized total density of states (TDOS) for all compositions, calculated using the optimized crystal structure, along with the partial DOS (PDOS) contribution from each element. The calculated total magnetic moment (*M*_total_), along with the individual moment from each element of the compounds, is presented in Table 1. As can be seen in Fig. 3, it is clear that Fe and Co provide a dominant contribution to the total magnetic moment, while Al shows a small negative moment. This negative moment indicates that, below the Fermi level (*E*_F_), the occupation of the minority spin channel (↓) of Al exceeds the majority spin channel (↑). The role of the Al atom (Z atom), however, cannot be overlooked in the alloy since the effective number of electrons in the d-orbitals can be tuned by the valence electrons of the Z atom, following the electron counting rule as discussed by Galanakis *et al.*^46^ The Slater–Pauling rule estimates the total magnetic moment (*M*_total_) of a compound based on the average valence electron number per formula unit (*N*_v_),^41^ given as

2 *M*_tot_ = (*N*_v_ − 24)

**Fig. 4:**
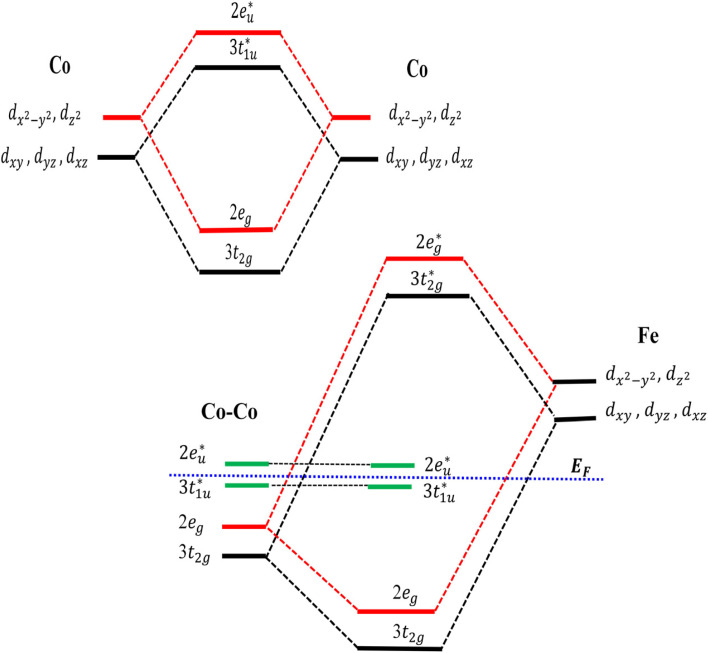
Schematic diagram of possible d–d hybridization of Co–Co and Co–Fe atoms' minority spin orbitals in Co_2_FeAl.

As Co has more valence electrons than Fe, the increase in Co content (*i.e.*, increasing *x* in Fe_2−*x*_Co_1+*x*_Al) is expected to cause a corresponding increase in *M*_total_, consistent with the Slater–Pauling rule. But when we look at the Fe-rich compounds, especially Co_1.25_Fe_1.75_Al and Fe_2_CoAl, the magnetic moment shows a clear deviation from the standard Slater–Pauling rule. As reported by Ahmad *et al.*^47^ and Aledealat *et al.*,^48^ the Fe_2_CoAl alloy exhibits an inverse XA structure due to its lower total energy compared to the L2_1_ structure, with space group *F*4̄3*m* and as for Co_1.25_Fe_1.75_Al, it exhibits a distorted cubic structure (tetragonal) with space group *P*4̄2*m* after relaxation, as tabulated in Table 1. For Fe_2_CoAl, *N*_v_ is 28; according to the SP rule (above), the expected *M*_total_ is 4*µ*_B_, but the calculated *M*_total_ is 5.139*µ*_B_. The SP rule is strictly valid only for the ordered cubic L2_1_ structure. Therefore, the observed deviations in *M*_total_ for the Fe-rich compounds are mainly attributable to their changes in crystal symmetry. These structural changes alter the electron band filling, leading to the minority spin channel holding fewer than the expected 12 electrons, and the majority spin channel, on the other hand, holds more than 16 electrons.^49^ The variation in Fe and Co concentrations in the alloy also induces a change in the spin polarisation at the Fermi level (*E*_F_). The spin polarisation, which quantifies the net spin asymmetry at *E*_F_, was calculated using the empirical formula^50^3
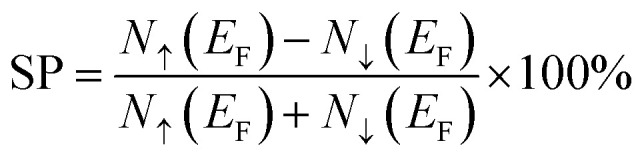
where *N*_↑_(*E*_F_) & *N*_↓_(*E*_F_) represent the spin-up (majority) and spin-down (minority) DOS at the Fermi level (*E*_F_), respectively. For Co_2_FeAl, the TDOS at *E*_F_ is dominated by the majority-spin channel (↑), contributing 3.79 states per eV, with only a small minority contribution of 0.41 states per eV, consistent with the previous study by Madhav *et al.*^24^ The minority-spin channel (↓) dominates at *E*_F_ for the Fe-rich compositions. As the Fe atoms are replaced by higher valence Co atoms, the system now has more electrons to fill in its available electronic states, causing a shift in the bands (Fig. 3). This substitution increases the total valence electron count, and the associated exchange splitting shifts the minority occupied states (also the majority unoccupied states) to higher energies. This enables the majority spin states to accommodate more electrons, altering the spin polarization at *E*_F_. This ultimately results in a gradual increase of the spin polarization, where the majority-spin channel now starts to dominate at *E*_F_, leading to positive polarization as Fe is replaced by Co atoms. Using the above equation, the calculated spin polarisation for Co_2_FeAl is 80.48%, which is the highest among all the compounds considered in this study. In half-metallic Co_2_FeAl, generally, each Fe and Al atom has eight Co atoms placed in an octahedral symmetry position as its first nearest neighbours, while each Co atom has four Fe and four Al atoms as its first nearest neighbours, forming a tetrahedral co-ordinated symmetry. The two crystallographically distinct Co atoms occupy Wyckoff positions at (1/4, 1/4, 1/4) and (3/4, 3/4, 3/4), and despite being second neighbors to each other, the Co–Co interactions play a dominant role in determining the electronic structure due to the high symmetry of the L2_1_ structure. By virtue of symmetry and transformations discussed by Galanakis *et al.*,^46^ the Co e_g_(d_*x*^2^–*y*^2^_ and d_*z*^2^_) and t_2g_(d_*xy*_, d_*yz*_ and d_*xz*_) orbitals are constrained to couple only with the e_g_ and t_2g_ orbitals at the other Co/Fe site. The interaction between the t_2g_ orbitals of Co–Co atoms forms a triple-degenerated bonding (t_2g_) orbitals at a lower energy level and antibonding 
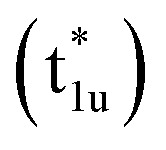
 orbitals at a higher energy level. Similarly, hybridization of the e_g_ orbitals results in bonding (e_g_) and antibonding 
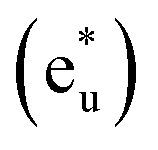
 orbitals; the coefficients associated with each orbital in Fig. 4 represent the degeneracy of that orbital. Moreover, the 3 × t_2g_ orbitals of the Co atoms interact with the t_2g_ orbitals of the Fe atom, forming three bonding orbitals below *E*_F_ and three antibonding orbitals at an energy above *E*_F_. The anti-bonding Co–Co orbitals (
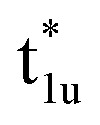
 and 
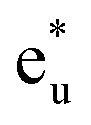
) are left uncoupled due to incompatible symmetry with the Fe d-orbitals, which are now called the non-bonding 
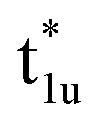
 and 
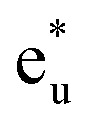
 orbitals located around *E*_F_. The absence of hybridization between these non-bonding orbitals and Fe d-orbitals leads to the splitting of states. This gives rise to an energy gap in the minority spin channel.

**Fig. 5:**
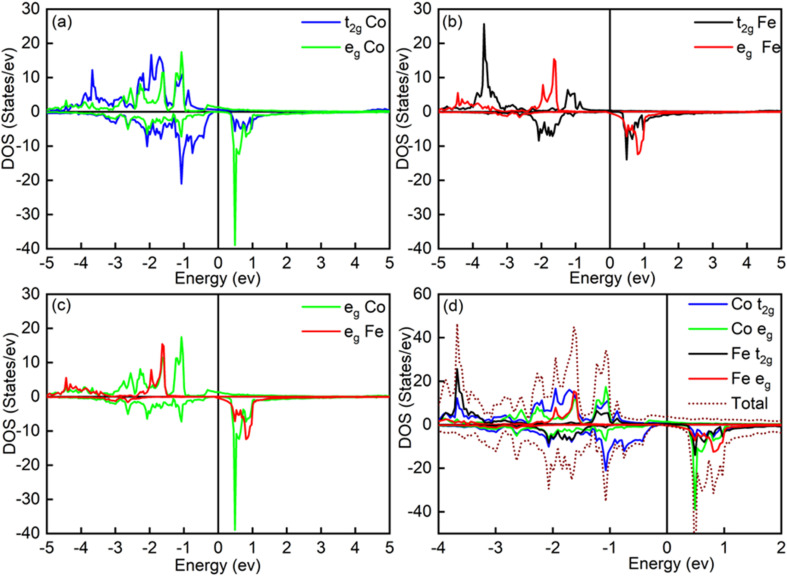
DOS of (a and b) t_2g_ & e_g_ orbitals of Fe and Co atoms, (c) t_2g_ & e_g_ orbitals of Co and Fe atoms, and (d) t_2g_ & e_g_ orbitals of Fe and Co atoms, and the total DOS of the Co_2_FeAl alloy.

**Fig. 6:**
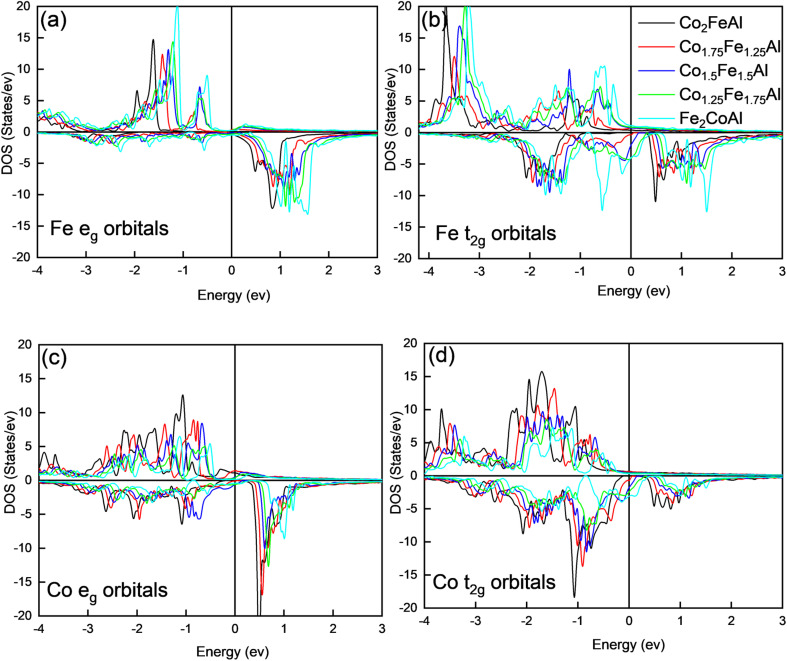
PDOS of Fe_2−*x*_Co_1+*x*_Al compounds (a and b) Fe e_g_ and t_2g_ orbitals & (c and d) Co e_g_ and t_2g_ orbitals.

In this study, Co_2_FeAl shows a pseudogap just below *E*_F_, indicating slight hybridization between the non-bonding Co orbitals (
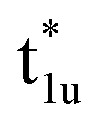
 and 
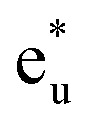
) and the Fe d-orbitals. The partial density of states (PDOS) for the Fe t_2g_-orbitals (Fig. 5) shows a broad minority spin gap at *E*_F_, while the Fe e_g_-orbitals exhibit a pseudogap nearly at *E*_F_. This distinct gap behavior observed in Fe orbitals is absent at the Co sites, where minority spin states remain finite near *E*_F_. These observations indicate that the minority states present below *E*_F_ are predominantly localized at the Co sites, with minimal hybridization between the non-bonding Co states and the Fe d-orbitals. With increasing Co concentration, there is a notable decrease in the minority spin channel DOS, particularly pronounced in the Fe-t_2g_ states, as can be seen in Fig. 6, and thus only for *x* = 1, *i.e.*, for Co_2_FeAl, a broad gap appears around *E*_F_ (Fig. 5). This reduction in the minority spin DOS around *E*_F_ signifies reduced hybridization between the orbitals, resulting from structural changes, and altered electronic configurations, which ultimately induce the observed changes in magnetic moment and the spin polarization as discussed above.

### 3.2 Experimental analysis

#### 3.2.1 Structural and morphological analysis

Fig. 8 presents the X-Ray Diffraction (XRD) pattern of the set of prepared alloys. The observed Bragg diffraction peaks at 2*θ* = 45°, 65° and 82° correspond to (220), (400), and (422) lattice planes of FCA^23^ and CFA^17^ phases.

**Fig. 7:**
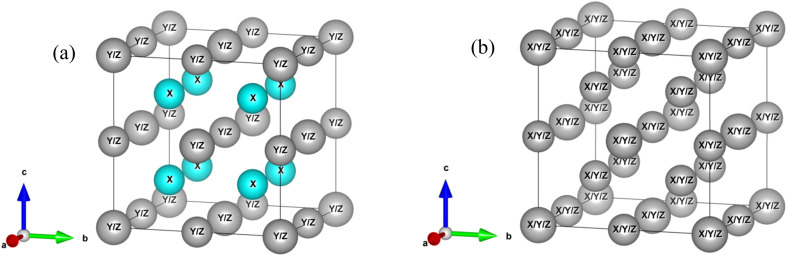
(a) B2 disorder and (b) A2 disorder Heusler structures.

In FHAs, the highly ordered L2_1_ phase is generally achieved when the constituent X, Y, and Z elements are in their ideal Wyckoff positions (Fig. 7) indicated by the presence of the (111) superlattice reflection. The absence of the (111) plane, with the presence of the (200) superlattice reflection, indicates the formation of a B2-type disordered structure, which is a partially disordered variant of the L2_1_ phase where the Y and Z elements randomly occupy each other's designated sites. The random distribution of the lattice sites and intermixing of the X, Y, and Z elements produce a fully disordered A2 phase. The random occupation of the lattice sites and intermixing of X, Y & Z elements give rise to a fully disordered A2 phase, which is indicated by the absence of both (111) and (200) superlattice reflections in the diffraction pattern.^17,51,52^ The principal reflections (220), (400), and (422) remain unaffected by the chemical ordering of atoms. However, with disorders, the intensity of the superlattice reflections (111) and (200) is either reduced or becomes negligible.^53^

**Fig. 8:**
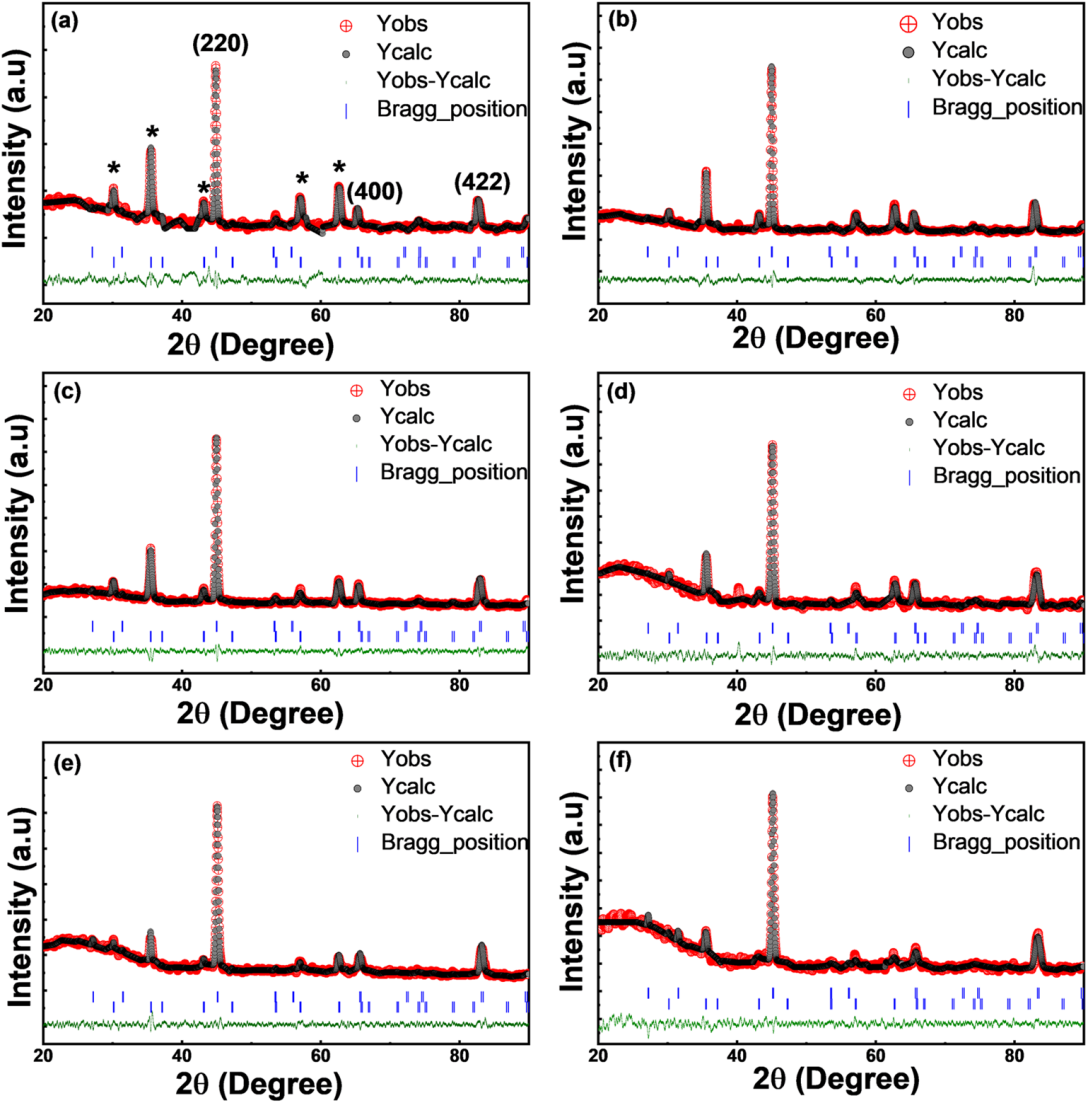
Rietveld refined XRD patterns of (a) Fe_2_CoAl, (b) Co_1.2_Fe_1.8_Al, (c) Co_1.4_Fe_1.6_Al, (d) Co_1.6_Fe_1.4_Al, (e) Co_1.8_Fe_1.2_Al, and (f) Co_2_FeAl.

**Fig. 9:**
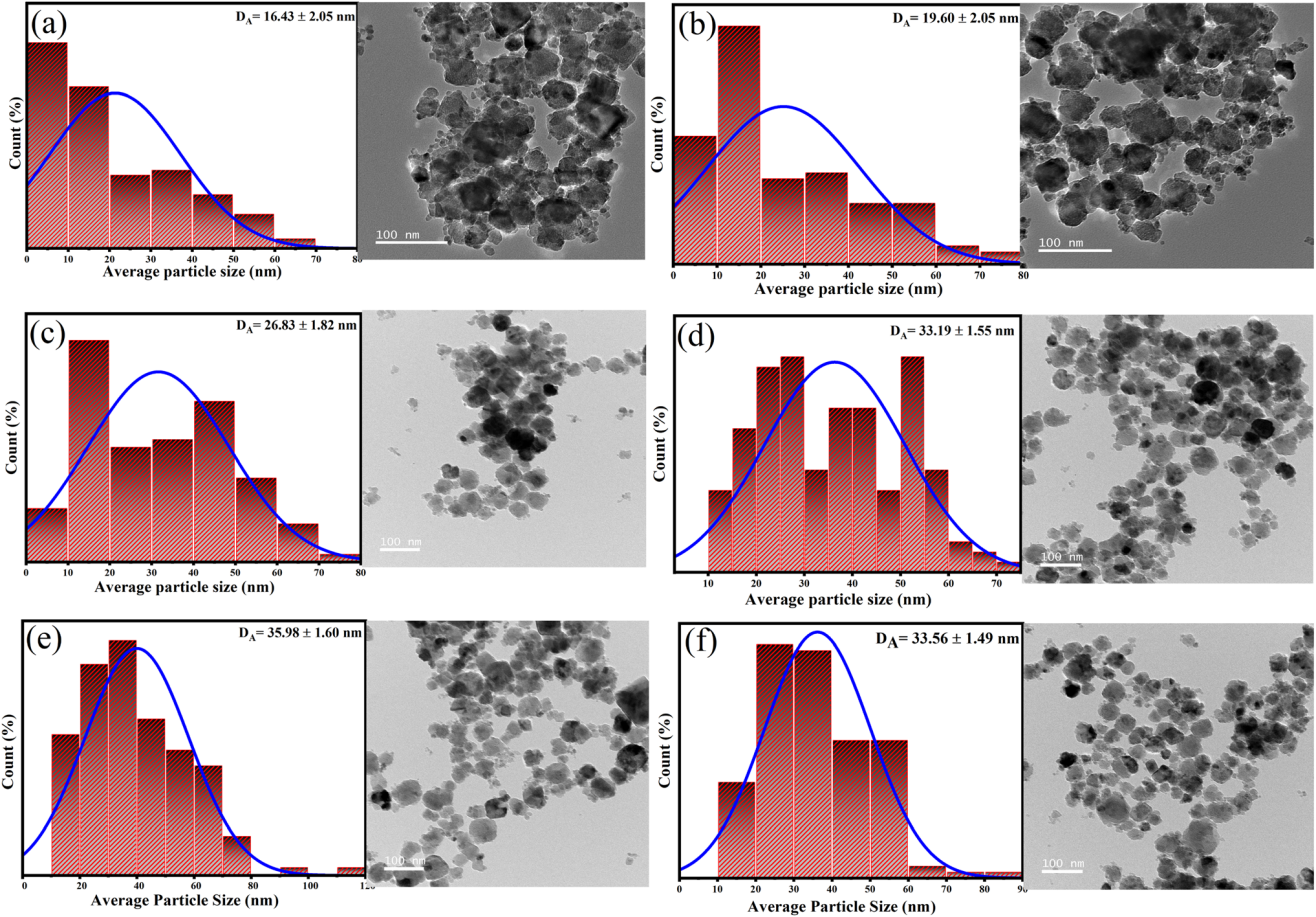
Histogram of average particle size distribution and TEM images of (a) Fe_2_CoAl, (b) Co_1.2_Fe_1.8_Al, (c) Co_1.4_Fe_1.6_Al, (d) Co_1.6_Fe_1.4_Al, (e) Co_1.8_Fe_1.2_Al & (f) Co_2_FeAl alloys.

**Fig. 10:**
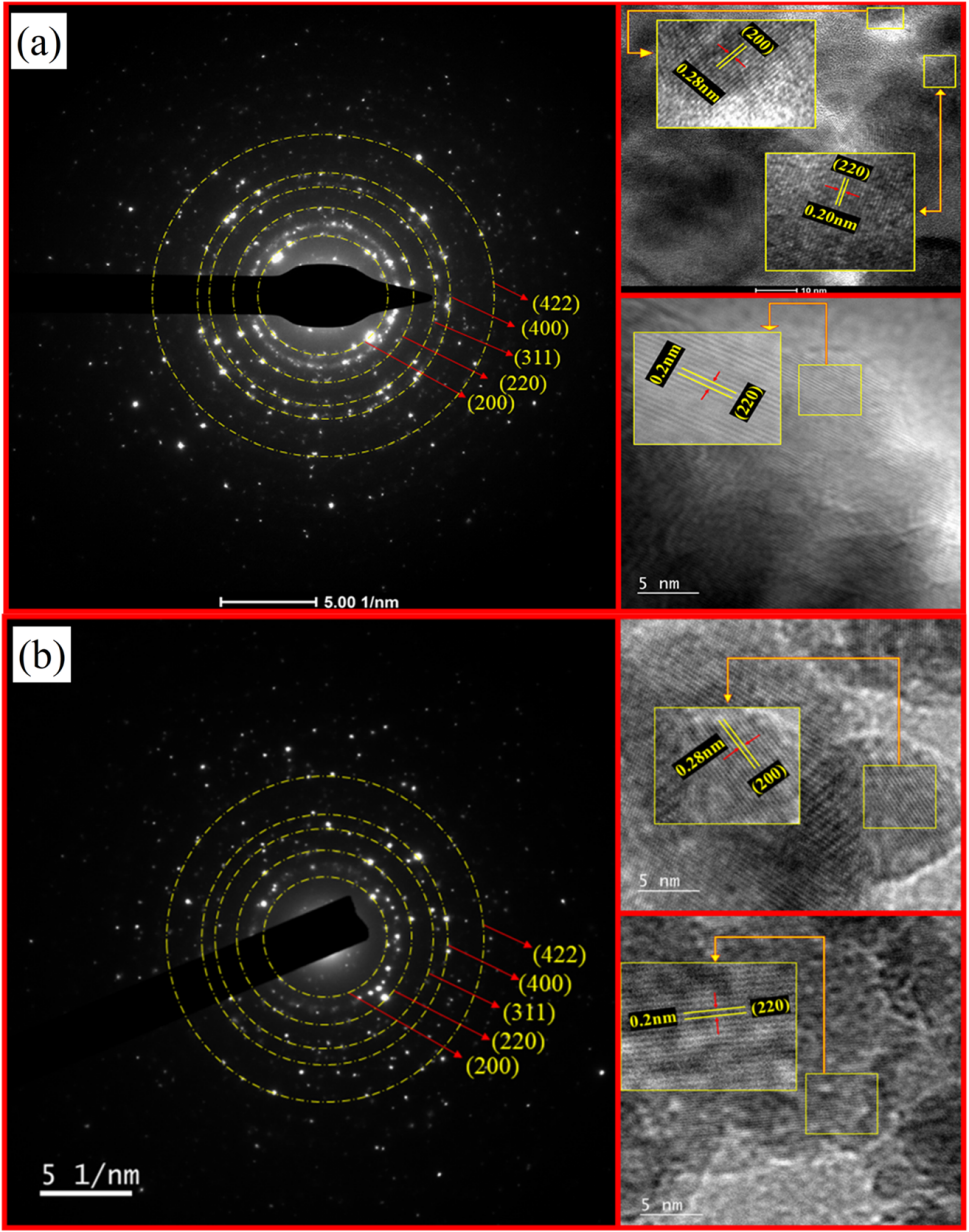
SAED pattern and HRTEM images of (a) Fe_2_CoAl and (b) Co_2_FeAl.

The weak (111) and (200) superlattice reflections are not observed in the XRD pattern of the prepared samples, indicating either the formation of an A2-type disordered structure,^54,55^ or a mixed phase of L2_1_, B2, and A2 type. Another reason for the absence of these reflections could be that the relative intensities of these peaks are usually very low and sometimes happen to be below the detection limits of the measurement.^56^ To resolve this issue, further structural analysis using the electron diffraction technique was performed. The Selected Area Electron Microscopy (SAED) pattern (Fig. 10) and the High-Resolution Transmission Electron Microscope (HRTEM) micrographs of FCA and CFA alloys show the presence of (200) reflection, which was not observed in the XRD pattern. The high-resolution image shows prominent lattice fringes confirming the high crystallinity of the particles. An interplanar spacing of 0.28 nm and 0.2 nm corresponding to the (200) and (220) planes, respectively, is observed. Other reflections from (220), (400), and (422) planes can also be seen in the SAED pattern, which is consistent with the XRD results. This authenticates the formation of a B2-type structure in the synthesized nanoparticles. The morphology, along with the fitted Gaussian profile histogram of the size-distribution of the particles (Fig. 9) and Table 2 presenting the average particle size between 16 and 35 nm, clearly confirms the formation of the nanoparticles. The size distribution obtained from the micrographs shows an increase in the average particle size with increasing Co concentration. In the same order, it can also be clearly seen that the particles are more homogeneous and possess a near-spherical shape. This delves back to the preparation of the nanoparticles. The pH and the amount of reducing agent were kept the same during the entire synthesis of Fe_2−*x*_Co_1+*x*_Al alloys; the only parameter varied during the synthesis was the Fe and Co content. It is known that, by regulating the OH^−^ ions in the solution and maintaining the other experimental parameters, different nanoparticle morphologies can be obtained,^57^ which highlights the crucial role of pH and the reducing agent in the microstructure and magnetic properties of the nanoparticles.^22^ The reduction potential of Co^2+^ ion (−0.28 V) and Fe^2+^ ion (−0.44 V) makes it easier for Co^2+^ to reduce to its metallic form than Fe^2+^ under the same experimental conditions. In samples with more Fe content, this slower reduction of Fe provides ample conditions for Ostwald ripening;^58^ as nucleation is non-uniform, this results in broader size distribution and morphological inhomogeneity. This inhomogeneity in the morphology can explain the discrepancy in the crystallite size and the average particle size for compounds with more Fe content.

**Table 2:** Calculated lattice constants, crystallite sizes obtained from XRD analysis, and average particle sizes determined from TEM

Compound	Lattice constant (Å)	Crystallite size (nm)	Average particle size (nm)
Fe_2_CoAl	5.702	27.75	16.43 ± 2.05
Co_1.2_Fe_1.8_Al	5.692	27.33	19.60 ± 2.05
Co_1.4_Fe_1.6_Al	5.679	26.03	26.83 ± 1.82
Co_1.6_Fe_1.4_Al	5.676	23.84	33.19 ± 1.55
Co_1.8_Fe_1.2_Al	5.674	23.53	35.98 ± 1.60
Co_2_FeAl	5.662	22.60	33.56 ± 1.49

The XRD pattern exhibits multiple secondary oxide peaks (marked by * in Fig. 8) corresponding to the cubic Fe_3_O_4_ phase (JCPDS card no. 75-1610).^59^ Multiphase refinement of the XRD patterns was done using the FullProf suite^58^ software, and the refinement yielded a *χ*^2^ value close to 1 for all the samples, thus showing that the model agrees with the experimental data (Fig. 8). The weight fraction of the primary and secondary phases for Fe_2−*x*_Co_1+*x*_Al as obtained from the refinement is 55.47% and 44.55% for *x* = 1, and with an increase in *x*, the primary phase increases with a maximum of 97.37%, while the secondary phase fraction reduces to 2.63% for *x* = 0.8. With decreasing Fe content and a simultaneous increase in Co content, there is a reduction in the intensity of the diffracted secondary peaks. Since the surface-state energy and charge density of Fe in its ferromagnetic state are higher than those of Co,^60^ this enhances the electron transfer, promoting oxidation in the Fe-rich compound. Using the Debye–Scherrer method,^58^ the crystallite size of the samples is obtained and is tabulated along with the lattice constants in Table 2. The decrease in the lattice constant with increasing Co content is attributed to the smaller atomic radius of Co (0.125 nm) compared to Fe (0.126 nm) atoms, while the decrease in the crystallite size (27 nm to 22 nm) is due to the Ostwald ripening as discussed above.

#### 3.2.2 Magnetic analysis

**Fig. 11:**
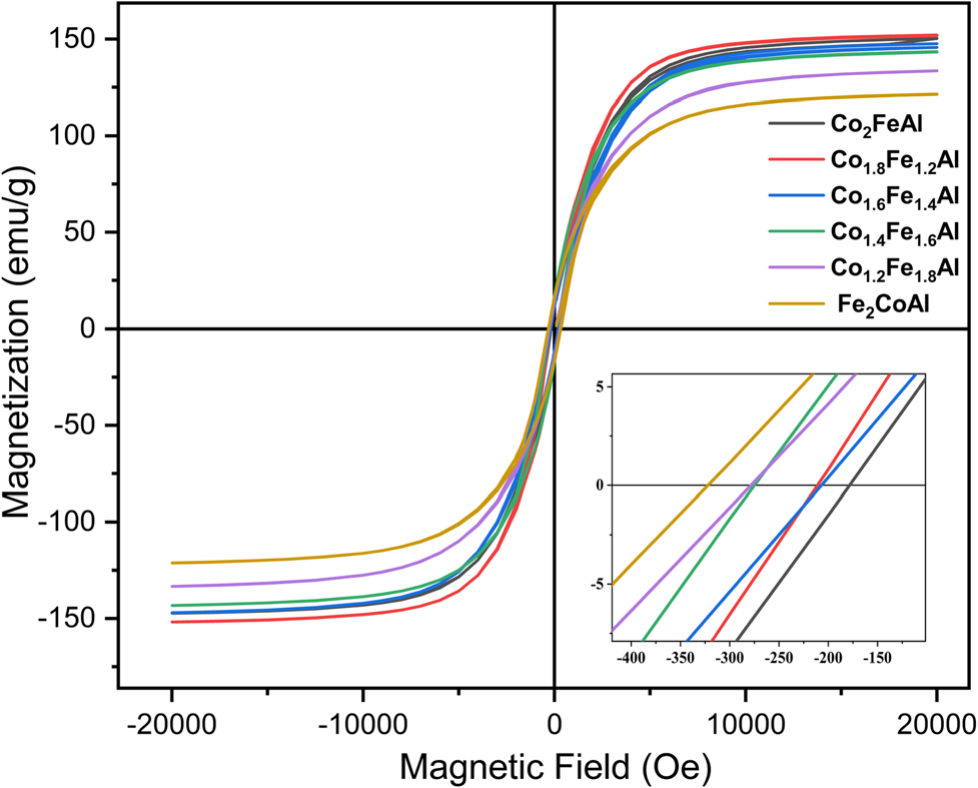
Magnetization as a function of the applied magnetic field (hysteresis loop) recorded at 300 K of Fe_2−*x*_Co_1+*x*_Al (*x* = 0, 0.2, 0.4, 0.6, 0.8 & 1) alloys.

Fig. 11 shows the field-dependent magnetization (*M*–*H*) curve of the Fe_2−*x*_Co_1+*x*_Al alloy series at 300 K. The observed hysteresis loop indicates that all compounds are ferromagnetic at room temperature. The saturation magnetization (*M*_s_) tends to increase with Co content and reaches a maximum of 152.51 emu g^−1^ (5.46*µ*_B_ f.u.^−1^) for the Co_1.8_Fe_1.2_Al composition, thereafter slightly reducing to 150.25 emu g^−1^ (5.40*µ*_B_ f.u.^−1^) for the Co_2_FeAl alloy. Experimentally determined moments are larger than the predicted L2_1_-based Slater–Pauling values of 4.0*µ*_B_ f.u.^−1^ and 5.00*µ*_B_ f.u.^−1^ for Fe_2_CoAl and Co_2_FeAl, respectively, but are marginally smaller than the theoretically calculated magnetic moments, as shown in Table 1. The Slater–Pauling prediction consistently follows mostly for ordered bulk L2_1_ Heusler structures, and thus, the reason for the deviation in the predicted moment can be attributed to the presence of chemical disorder (B2 type) in the nanostructured alloys, as reported by A. Ahmad *et al.*^61^ and validated by the XRD data and the SAED pattern. The theoretical calculations correspond to the ideal chemically ordered phase and thus serve as a reference framework and a starting point for understanding the system's fundamental electronic and magnetic properties. Site/antisite disorder, impurities/secondary phases, finite-temperature effects, and morphological and compositional variations also affect the deviation between the experimental and theoretical values of the magnetic parameters.

A comprehensive comparison of the magnetic properties, including saturation magnetization (*M*_s_), coercivity (*H*_c_), and the effective anisotropy constant (*K*_eff_) obtained from the Law of Approach to Saturation (LAS)^62^ fitting for Fe_2−*x*_Co_1+*x*_Al alloys, is presented in Table 3. A clear trend is observed in which decreasing the Fe concentration enhances the saturation magnetization while reducing the coercivity. The interplay of the following key factors accounts for the observed magnetic behavior. The presence of the secondary phase significantly affects the alloy's coercivity. The secondary phase Fe_3_O_4_, which is ferrimagnetic in nature, interacts in an antiparallel manner to the main ferromagnetic phase of the alloy. The interfacial exchange coupling between the dominant ferromagnetic phase (Fe_2_CoAl/Co_2_FeAl) and the ferrimagnetic Fe_3_O_4_ phase enhances domain-wall pinning, thereby suppressing domain-wall motion and increasing the coercivity.^63^ Accordingly, the Fe_2_CoAl alloy exhibits the highest coercivity due to its largest Fe_3_O_4_ volume fraction. With increasing Co content, the Fe_3_O_4_ fraction decreases, weakening the interfacial exchange coupling and resulting in a significant reduction in coercivity from 322.33 Oe for Fe_2_CoAl to 177.91 Oe for Co_2_FeAl. Furthermore, larger nanoparticle size reduces the fraction of surface atoms with broken exchange bonds, thereby decreasing surface spin disorder and increasing the net magnetization.^63^ The increase in magnetization with particle size (Table 2) can be attributed to the surface-to-volume (S/V) ratio of the nanoparticles. Consequently, the observed increase in magnetization with increasing particle size (Table 2) can be attributed to the decreasing surface-to-volume (S/V) ratio, which reduces surface spin disorder and allows a larger fraction of interior spins to contribute to the total magnetization. In contrast, smaller particles with higher S/V ratios favor a higher volume fraction of surface oxides,^64^ further diminishing magnetization. Additionally, the enhancement of the saturation magnetization with increasing Co content correlates with the experimentally observed lattice contraction, consistent with strengthened magnetic exchange interactions. Although lattice contraction can enhance transition metal (TM)–Al (Co–Al and Fe–Al) hybridization, which may reduce local magnetic moments in highly ordered Heusler phases, the alloys studied here exhibit partial chemical disorder as confirmed by XRD. In these predominantly disordered (B2/A2-type) alloys, the increased number of nearest-neighbor Co–Co and Co–Fe pairs strengthens d–d exchange interactions, which dominate TM–Al hybridization and result in an overall increase in the saturation magnetization.

**Table 3:** Magnetic properties of Fe_2−*x*_Co_1+*x*_Al (*x* = 0, 0.2, 0.4, 0.6, 0.8 & 1) alloys recorded at 300 K

Compound	*M*_s_ (emu g^−1^)	Moment (*µ*_B_ f.u.^−1^)	*H*_c_ (Oe)	*K*_eff_ × 10^6^ (erg cm^−3^)	Relative fitting error
Fe_2_CoAl	121.26	4.29	322.33	1.00	0.9978
Co_1.2_Fe_1.8_Al	133.56	4.74	278.19	1.11	0.9979
Co_1.4_Fe_1.6_Al	143.21	5.10	277.79	1.11	0.9968
Co_1.6_Fe_1.4_Al	146.66	5.24	206.88	1.02	0.9981
Co_1.8_Fe_1.2_Al	152.51	5.46	211.83	0.95	0.9976
Co_2_FeAl	150.25	5.40	177.91	1.06	0.9977

Building on this, the effective magnetic anisotropy, *K*_eff_, was also investigated, as it is a fundamental parameter that determines the stability of magnetic moments against thermal fluctuations and applied fields, influencing both coercivity and overall magnetic performance. In the present study, *K*_eff_ was estimated using the Law of Approach to Saturation (LAS) fitting of high-field magnetization data, which provides a quantitative measure of intrinsic anisotropy.

The LAS fitting for cubic polycrystalline ferromagnets was performed using the following equation:
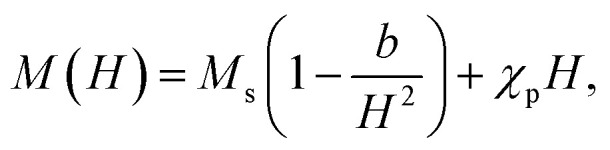
where *M*_s_, *b* and *χ*_p_ are the saturation magnetization, anisotropy-related coefficient and high-field susceptibility contribution. At a high-field, the magnetization becomes robust against domain wall motions and mainly changes due to the spin rotation against the anisotropy energy. The fitting range was above 5 kOe.

And the effective anisotropy constant *K*_eff_ was obtained using the equation
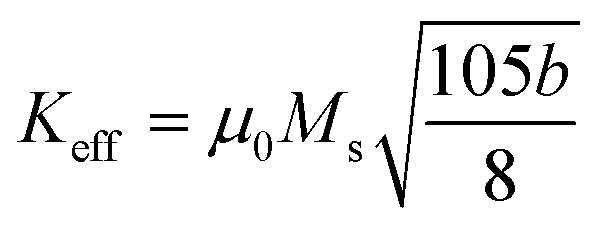


The estimated *K*_eff_ values are nearly constant across the different compositions, indicating that magnetic anisotropy is largely insensitive to particle size or Co content. This behavior suggests that *K*_eff_ is primarily governed by chemical disorder (B2 type), which introduces local site-specific strains that modify the orbital contribution to the magnetic moment. While the magnetocrystalline anisotropy of fully ordered L2_1_ structures is generally weak, B2 disorder enhances *K*_eff_ by breaking cubic symmetry and increasing spin–orbit coupling at anti-site positions.^27,65,66^ The anisotropy extracted from the LAS fitting corresponds to the effective anisotropy constant, which contains contributions from anisotropies arising from disorder, strain, and defects, alongside magnetocrystalline anisotropies. The origin of magnetocrystalline anisotropy is the spin–orbit coupling, *i.e.*, *H*_SOC_ = *ξLS*, where *ξ* is the coupling constant. Any changes or modifications in the symmetry of the crystal are bound to influence the anisotropy energy, since the SOC links the spin alignment with the orbital electronic environment.^67^ Slight variations in the estimated *K*_eff_ can be attributed to local strains and disorder-induced modifications of the orbital moment, further confirming the dominant role of B2 disorder in controlling magnetic anisotropy.

In addition to this intrinsic contribution, extrinsic effects such as nanoparticle shape anisotropy and interfacial anisotropy at the Fe_2_CoAl–Co_2_FeAl/Fe_3_O_4_ interfaces also contribute to the overall anisotropy, with their influence being more pronounced in Fe-rich compositions where the oxide volume fraction is highest. Collectively, these results demonstrate that the magnetic behavior of the present alloys is governed by a complex interplay of chemical disorder, exchange interactions, and interfacial effects.

## 4 Conclusion

DFT calculations reveal that cobalt-rich compositions exhibit a pseudogap in the minority spin channel and a high spin polarization and follow the Slater–Pauling rule, while iron-rich compositions show structural distortions, enhanced minority-spin hybridization, and thus reduced/negative spin polarization. The hydrothermally synthesized nanoparticles predominantly adopt the B2 type partial disorder, with particle sizes ranging from 16 to 35 nm, lattice contraction occurring with increasing cobalt content, and secondary ferrimagnetic phases being more prominent in iron-rich alloys due to their higher oxidation affinity. The nanoparticles display strong room-temperature ferromagnetism, with *M*_s_ reaching a maximum of 152.51 emu g^−1^ (5.46*µ*_B_ f.u.^−1^) for the Co_1.8_Fe_1.2_Al composition, exceeding the Slater–Pauling predictions due to disorder, enhanced interactions, and reduced surface spin canting in larger particles as oxide-induced surface interaction weakens. Collectively, this study underscores the influence of composition, atomic disorder, morphology, and secondary phases on the magnetic behavior of the alloys, yet affirms the potential for tailored spintronic applications through compositional and synthesis control. Beyond the specific Fe–Co–Al system, this work establishes a framework for rational design of functional intermetallic nanoparticles by integrating computational predictions with optimized low-temperature synthesis strategies.

## Conflicts of interest

There are no conflicts to declare.

## Data Availability

The data that support the findings of this study are available within the article.
